# Improving Fat Graft Survival Using Soluble Molecule Preconditioning

**DOI:** 10.3390/biom15040526

**Published:** 2025-04-03

**Authors:** Nabil Amraoui, Isabelle Xu, Jorge Robles Cortés, Chanel Beaudoin Cloutier, Julie Fradette

**Affiliations:** 1 Regenerative Medicine Division, CHU de Quebec-Université Laval Research Center, Quebec, QC G1J 1Z4, Canada; nabil.amraoui@umontreal.ca (N.A.);; 2 Department of Surgery, Faculty of Medicine, Université Laval, Quebec, QC G1V 0A6, Canada; isabelle.xu.1@ulaval.ca (I.X.); jorge.robles-cortes.1@ulaval.ca (J.R.C.); 3 Centre de Recherche en Organogenèse Expérimentale de l’Université Laval/LOEX, 2255 Vitré Avenue, Quebec, QC G1J 5B3, Canada

**Keywords:** fat grafting, adipose tissue, fat graft survival, preconditioning, volume retention, oxidative stress, angiogenesis, adipogenesis, antioxidant

## Abstract

Fat grafting is widely used in plastic surgery to correct soft tissue deformities. A major limitation of this technique is the poor long-term volume retention of the injected fat due to tissue remodeling and adipocyte death. To address this issue, various optimizations of the grafting process have been proposed. This scoping review focuses on preclinical and clinical studies that investigated the impact of various classes of soluble molecules on fat grafting outcomes. Globally, we describe that these molecules can be classified as acting through three main mechanisms to improve graft retention: supporting adipogenesis, improving vascularization, and reducing oxidative stress. A variety of 18 molecules are discussed, including insulin, VEGF, deferoxamine, botulinum toxin A, apocynin, N-acetylcysteine, and melatonin. Many biomolecules have shown the potential to improve long-term outcomes of fat grafts through enhanced cell survival and higher volume retention. However, the variability between experimental protocols, as well as the scarcity of clinical studies, remain obstacles to clinical translation. In order to determine the best preconditioning method for fat grafts, future studies should focus on dosage optimization, more sustained delivery of the molecules, and the design of homogenous experimental protocols and specific clinical trials.

## 1. Introduction

Since its origins dating back to the 1800s, when a German surgeon named Gustav Neuber first transplanted adipose tissue into an orbit, fat grafting has become one of the most common procedures in plastic and reconstructive surgery [1]. Lipotransfer procedures are mainly used to correct volume and contour abnormalities for reconstructive purposes but have also proven useful in reducing post-mastectomy pain [2] and even contributing to peripheral nerve surgery outcomes [3,4]. This technique is not only a powerful and versatile reconstructive tool but also has many significant advantages: simplicity, easy access, low risk, low cost, and non-immunogenicity [3]. However, the main challenge associated with lipotransfer is the poor volume retention of fat grafts, with current literature reporting a 20 to 80% resorption rate in the following years after transplantation [5]. The literature describes three areas in the fat graft: peripheral (where surviving adipocytes are found), intermediate (where the inflammatory process takes place), and central (the necrotic zone, where adipocytes are too far to benefit from diffusion) [6]. Many theories have been proposed to explain fat graft survival. In the *graft replacement theory*, volume maintenance depends mainly on the regeneration of fat tissue by the donor cells present in the graft itself (namely, adipocytes and adipose-derived stem cells from both the recipient site and the graft). More precisely, the *host cell replacement theory* suggests that adipose tissue undergoes necrosis in the initial phase of transplantation and is gradually replaced by cells from the recipient within the first months after grafting. Following this logic, other studies focused on molecules that either promote adipogenesis or reduce oxidative stress, all with the final objective of promoting the survival and function of the replacing cells. The *graft survival theory* is the most widely accepted and argues that the survival of fat grafts depends on the survival of the transplanted adipocytes [7]. These adipocytes survive because of the revascularization of the graft from the recipient site [8]. Thereby, many molecules promoting vascularization have been investigated to increase fat graft survival.

It is thus crucial to find ways to optimize fat grafting outcomes and reduce the need for repetitive interventions. Many strategies aimed at improving fat graft survival have been investigated throughout the years, with researchers trying to optimize the three main technical steps of fat grafting: harvesting, processing, and injection [9,10,11]. An interesting avenue for enhancing fat graft retention is the pretreatment of said fat with different additives during the processing phase before injection.

**Figure 1:**
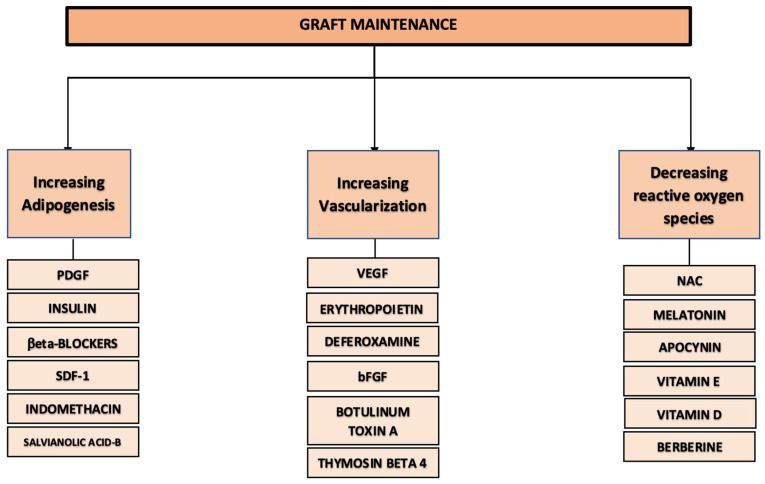
Main mechanisms of fat graft survival improvement mediated by types of soluble molecules. bFGF: basic fibroblast growth factor; NAC: N-acetylcystein; PDGF: platelet-derived growth factor; SDF-1: stromal cell-derived factor-1; VEGF: vascular endothelial growth factor.

In this manuscript, we review the most studied and promising soluble molecules that may help improve fat graft survival and volume retention, ranging from growth factors to neurotoxins and antioxidants. These molecules act on the three main biological processes determining adipocyte survival, maintenance, and regeneration after transplantation, namely, adipogenesis, vascularization, and oxidative stress (Figure 1). We first present the advances made toward the stimulation of adipogenesis since adipocytes and their precursors are the foremost cellular targets of molecular preconditioning. Then, the impact at the tissue level is described since supporting the cells through the surrounding vascular processes has been the most commonly studied modality in molecular preconditioning. Finally, we report on the newer studies focusing on the benefit of using soluble molecules able to globally reduce the impact of oxidative stress.

## 2. Materials and Methods

In March 2025, a retrospective review of the published data was conducted on PubMed, Cochrane, and Clinicaltrials.gov using the following keywords:

(*Molecule name*) AND/OR (fat graft OR fat grafting OR lipotransfer OR fat transfer OR lipograft OR lipofilling OR fat transplantation OR autologous fat OR fat graft) AND (volume retention OR volume maintenance OR graft retention OR viability OR vascularization OR retention OR Maintenance OR survival OR atrophy OR apoptosis OR loss OR inflammation OR pretreatment OR Mortality OR adipose tissue OR adipocyte OR adipose cells).

Articles relating to any soluble molecule used to improve fat graft survival were included, without restriction on the year of publication. Some manuscripts were, however, excluded based on the following exclusion criteria:

(1) Articles describing only graft survival enhancement techniques that did not involve growth factors/molecules;
(2) Reviews and meta-analyses;
(3) Studies combining molecules and cell-based therapy.

All articles of potential interest were collected, and titles, abstracts, and/or full articles were reviewed for eligibility and relevance. Some manuscripts dealt with more than one molecule of interest, but in total, 48 articles were selected for inclusion in the present review. Data were extracted and compiled in Appendix A, Appendix B and Appendix C (Table A1, Table A2 and Table A3). These tables describe the experimental designs and main findings of a wide range of studies, from animal models to clinical trials.

## 3. Discussion

### 3.1. Stimulating Adipogenesis

In the early stages following fat grafting, adipose tissue undergoes necrosis and massive cell death due to the lack of appropriate vascularization, which explains why only a portion of the grafted tissue survives [12]. Following the *graft replacement theory,* in which grafted adipose tissue is gradually replaced by cells from the graft and recipient sites [13], many studies have been focusing on the promotion of adipogenesis to improve graft survival. Of course, adipogenesis is a cellular process interdependent with the surrounding angiogenic cues, but six main types of molecules have been reported to act on this process, during which a precursor/stem cell becomes a lipid-filled mature adipocyte (Table A1, Appendix A).

#### 3.1.1. Platelet-Derived Growth Factor (PDGF)

PDGF is a potent stimulator of mesenchymal stem cell (MSC) proliferation and promotes angiogenesis in vivo, thus being able to improve wound healing [14]. Importantly, it has also been shown to stimulate the differentiation of murine preadipocytes and to prevent apoptosis [15,16]. Fontdevila et al. investigated the impact of PDGF mixed with platelet-rich plasma (PRP) on the survival of human autologous fat grafts used to treat patients with facial lipodystrophy secondary to human immunodeficiency virus (HIV) infection [17] (Table A1). The study showed no difference in volume retention between the PDGF-treated grafts and the control ones, concluding the possible ineffectiveness of PDGF treatment of fat grafts in HIV lipodystrophy cases [17]. Despite this rather discouraging conclusion, one must take some factors into consideration: PRP’s effect, the time since the diagnosis of lipoatrophy between the different patients, and the fact that HIV (and associated antiretroviral treatment) can cause adipose tissue dysfunction and inflammation [18]. These factors thus make it difficult to generalize this conclusion to non-HIV patients. Importantly, the study used free PDGF mixed with PRP. PDGF, however, has a short half-life of two minutes when injected intravenously [19]. It can be easily cleared by blood circulation [20]. Therefore, different delivery systems, such as microspheres or hydrogels, have been explored to ensure the controlled delivery of PDGF [20,21]. Craft et al. used gelatin microspheres as a continuous delivery system of PDGF and mixed it with human lipoaspirate before injecting it into the scalp of mice [22]. The treatment improved graft survival, promoted the maintenance of the adipocyte’s architecture, and increased the number of preadipocytes [22]. Thereby, PDGF improved the survival and quality of grafts by stimulating adipogenesis and preventing graft degeneration into fibrotic tissue. Interestingly, this study also included an experimental group receiving free PDGF, which showed that PDGF without a delivery system such as microspheres is significantly less efficient, even failing to show significant differences from control groups [22]. Thus, PDGF is promising, but further human-based studies are needed and should include delivery systems to ensure more stable and reliable local concentrations of PDGF. Paradoxically, PDGF in higher concentrations than those used in Craft’s study has been shown to have an inhibitory effect on the differentiation of murine preadipocytes in vitro [23]. Therefore, a dose–response curve needs to be investigated to determine optimal outcomes in the context of fat grafting.

#### 3.1.2. Insulin

Insulin acts as a key factor of adipogenesis by inducing the differentiation of precursors into adipocytes [24]. More precisely, the binding of insulin to its receptors leads to an increase in adipocyte glucose uptake by mobilizing glucose transporter type 4 (GLUT4) [25] and to an accumulation of lipids into adipocytes through the activation of lipoprotein lipase [26]. These processes ultimately lead to adipogenesis and adipocyte hypertrophy. In vivo, it has been reported that the sustained administration of insulin in the abdominal walls of rodents induced de novo adipose tissue formation [27]. Thus, it was suggested that insulin may have the potential to help graft retention by promoting adipose tissue regeneration. Hong et al. observed increased graft retention from 15% to 41% when using a transfer medium containing insulin during the lipotransfer procedure [28] (Table A1). The sustained delivery of insulin via polyethylene glycol (PEG) microspheres also mediated an increase in the volume maintenance of fat grafts when compared to untreated grafts, but this improvement did not significantly differ from treatment with other agents, such as bFGF and insulin-like growth factor-1 (IGF-1) [29]. The graft composition did differ depending on the agent used, with insulin increasing the number of adipocytes, suggesting that insulin may be better for enhancing adipogenesis [29]. Surprisingly, many studies reported disappointing results following insulin treatment. A 2019 study by Okyay et al. showed no significant improvement in the maintenance of fat grafts soaked for five minutes in an insulin solution before transplantation [30]. However, the soaking time may not have been long enough. Lu et al. preferred mixing insulin directly with fat before its injection and obtained limited results since insulin improved weight retention but also increased fibrosis and necrosis [31]. Similar outcomes were obtained by Ayhan et al., where insulin-treated grafts had better volume retention and more adipocytes but also featured considerable levels of inflammation [32]. Other studies from 1988 and 1994 again showed limited beneficial results, with no histopathologic differences between insulin-treated grafts and controls [33,34]. However, only qualitative assessments were made, and blood was not washed from fat grafts, which can increase fat necrosis. In contrast, some studies showed good outcomes with insulin when combined with other agents. In 2012, Cervelli et al. showed that PRP increased the proliferation of adipose-derived stem/stromal cells (ASCs) in vitro and potentiated insulin-induced adipogenic differentiation [35]. In humans, the best graft retention was obtained when insulin and PRP were used at the same time [35]. Thus, the two agents seem to have a synergistic effect, with PRP acting on increasing proliferation while insulin promotes intracytoplasmic lipid accumulation. Another study performed on rodents revealed that insulin treatment improved graft maintenance (75%) but not as much as erythropoietin (EPO) treatment (85%) [36]. However, when the two agents were mixed, the grafts had the best volume maintenance, reaching 95% [36]. Thereby, insulin could be a good option, but its effect may need to be potentiated by other agents. In addition, doses and mixing ratios of insulin with other molecules still need to be investigated.

#### 3.1.3. Beta-Blockers

β-adrenergic signaling has been shown to regulate MSC adipogenesis in a murine model [37]. In fact, activation of β-adrenergic receptors decreased adipogenic differentiation of MSCs, while antagonists promoted adipogenesis [37]. Furthermore, lipolysis of adipose tissue occurs through activation of β_1_-adrenergic receptors [38]. Following this logic, beta-blockers should support adipogenesis and adipocyte metabolic activities. A murine study conducted by Ayhan et al. showed that fat grafts mixed with metapyrolol, a selective β_1_ blocker, had less resorption and more surviving adipose tissue [32] (Table A1). In contrast, a more recent study using another β-blocker (metoprolol) failed to show a beneficial impact of the molecule on the volume retention of fat grafts [30]. However, metoprolol-treated grafts showed improved adipocyte viability compared to control and grafts treated with insulin [30]. In 2023, the same team conducted another murine study to investigate the effect of the metoprolol concentration on fat grafts [39]. The experiment showed that grafts soaked in metoprolol solution had better tissue viability, higher vascularization, and lower fibrosis compared to controls. Furthermore, this beneficial effect seems to be dose-dependent, with higher concentrations of metoprolol giving better results [39]. However, fat graft retention was not measured in the study. Thereby, beta-blockers may be an interesting avenue for fat graft improvement, but the type of molecule used, as well as dosages, need to be further studied.

#### 3.1.4. Other Molecules of Interest Impacting Adipogenesis and Adipocytes

Other less-studied molecules should also be mentioned. SDF-1 is a chemokine known to attract stem cells via its attachment to the CXCR4 receptor. It has proven useful in promoting heart, brain, and skin wound healing [40,41,42], and thus, its effect on fat graft survival has been investigated. In 2012, Hamed et al. conducted a study in which diabetic mice were injected with autologous lipoaspirate mixed with SDF-1 or phosphate-buffered saline (PBS) [43] (Table A1). While regular grafts were almost completely resorbed after 15 weeks in diabetic mice, SDF-1 treated grafts had better weight and volume retention that were, in fact, comparable to those of grafts performed in non-diabetic mice. This increased retention was associated with a higher migration of endothelial progenitor cells (EPCs) inside and outside of the grafts, suggesting a systemic effect of locally delivered SDF-1 [43]. Decreased cell apoptosis of fat grafts and increased plasma levels of VEGF were also noticed in SDF-1-treated mice [43].

Indomethacin, a non-steroidal anti-inflammatory drug, has been shown to promote adipogenesis in stem cells in vitro [44]. Indeed, studies found that the molecule up-regulates the expression of adipogenic genes [44]. Zhan et al. investigated its effect on fat graft retention in nude mice and found that the drug improved volume retention compared to lipoaspirate alone (Table A1). It also improved cell viability and promoted the expression of adipogenic genes by ASCs in vitro [45]. However, no effect was found on vascularization, suggesting that the mechanism of action of indomethacin is mainly through adipogenesis stimulation.

Salvianolic acid-B (Sal-B) is a molecule found in *Salvia miltiorrhiza*, a traditional chinese medicinal plant. Studies have shown that it improves the expression of adipogenic transcription factors in preadipocytes [46]. Sun et al. conducted two studies aimed at investigating the impact of Sal-B on fat graft retention and adipogenesis (Table A1). In vitro, Sal-B accelerated the adipogenic differentiation of ASCs, showing that the molecule could be beneficial in fat graft retention through its action on adipogenesis stimulation [47]. Sal-B also reduced macrophage polarization and inflammation in vitro, suggesting an anti-inflammatory effect of the molecule [48]. On graft retention, both studies showed an improved volume maintenance of fat grafts in a murine model [47,48]. More precisely, Sal-B grafts had 59.36% volume retention, compared to 15.51% for grafts treated with saline [47].

### 3.2. Improving Vascularization

Adequate blood perfusion has been proven essential to tissue maintenance after lipotransfer, with adipocytes dying as soon as the first day of ischemia [12,49]. In the early stages of transplantation, adipocyte survival depends on the plasmatic diffusion of nutrients from the surrounding tissues until adequate vascularization takes over [12]. According to the *graft survival theory*, once vascularization is established, grafted adipocytes can survive in the long term. Stimulating early vascularization and angiogenic processes through soluble mediators could, therefore, decrease the necrotic area and help maintain the graft volume. This field has been widely studied through various modalities. The impact of six molecules of interest is described below and summarized in Table A2, Appendix B.

#### 3.2.1. Vascular Endothelial Growth Factor (VEGF)

VEGF is an angiogenic growth factor that exists in four isoforms, VEGF165 being the most physiologically active and most thoroughly studied [50]. VEGF is known to be the main stimulatory factor for neovascularization by stimulating endothelial cell proliferation and migration, as well as promoting vasodilatation and making capillaries more permeable [50,51,52]. However, VEGF’s short half-life (approximately 30 min) is an obstacle [53]. Predictably, the use of VEGF without a delivery system led to rather disappointing results. Hamed et al. showed no significant difference in the weight and volume of grafts treated with VEGF when compared to controls treated with PBS, even with repeated injections every three days [54] (Table A2). Interestingly, different types of microspheres have been used for the sustained delivery of VEGF. Chung et al. used polylactic-co-glycolic acid (PLGA) microspheres containing VEGF to successfully improve graft sustainability and vascularization in a murine model [55]. Other types of VEGF-encapsulating microspheres have also been explored, with studies mixing fat grafts with chitosan, polylactide acid (PLA), or calcium alginate microspheres, all leading to higher volume retention and vascularization [56,57,58,59]. It may be important to note that, at high concentrations, chitosan nanospheres showed cytotoxic effects on adipocytes [57]. Also, calcium alginate microspheres were not completely absorbed in some grafts, meaning that the increase in volume and weight retention may not only be due to improved graft viability or regeneration but also to this material itself. VEGF microspheres can also be used in recipient site preconditioning. For example, as described by Topcu et al., enhanced graft retention was achieved by injecting calcium alginate VEGF microspheres at the recipient site 21 days prior to lipotransfer [58]. Globally, these studies indicate that many strategies aiming at providing early sustained supplementation of VEGF in the grafts can improve the viability of adipocytes by promoting faster vascularization. Microspheres appear to be a promising approach and should be studied more extensively.

#### 3.2.2. Erythropoietin (EPO)

EPO is the main stimulating agent of erythropoiesis. It has also been described as a key regulator of mesenchymal stem cell endothelial differentiation, making it a good candidate for graft improvement therapy [60]. It has been shown to have an anti-apoptotic effect on endothelial cells in addition to inducing a pro-angiogenic phenotype in vitro [61,62,63]. EPO also plays an indirect role in vascularization by promoting the expression of VEGF, which, as mentioned earlier, plays a pivotal role in angiogenesis [64]. This could explain EPO’s effectiveness in promoting cerebral, bone, and myocardial repair through angiogenesis in animal models undergoing ischemic damage [65,66,67]. In vitro, EPO has also been able to improve ASC migration but had no significant impact on their metabolic activity [68]. Thus, EPO might support graft retention through the promotion of vascularization. Olaru et al. performed autologous fat grafts in Wistar rats after mixing the adipose tissue with EPO and/or insulin [36] (Table A2). Compared to untreated grafts, EPO led to increased vascularization and volume maintenance (85%), even though the best outcome was with the mix of EPO and insulin (95%), suggesting a synergistic effect of these molecules [36]. Another study by Hamed et al. showed that EPO increased the maintenance of human fat grafts in a murine model through the stimulation of angiogenesis [54]. This neovascularization occurred due to EPO’s stimulation of a cluster of proangiogenic chemokines, including VEGF, bFGF, and insulin-like growth factor 1 (IGF-1) [54]. Thus, EPO seems to stimulate the expression of various growth factors that act in synergy, which makes it an interesting path of investigation. Indeed, EPO has been used safely in humans for years for the treatment of conditions like anemia. Further research is, however, needed for dose optimization before entering clinical trials in the context of fat grafting.

#### 3.2.3. Deferoxamine (DFX)

DFX is an iron-chelating drug that shows great promise in tissue regeneration. In addition to its antioxidant and immunomodulatory benefits, DFX has a pro-angiogenic effect by increasing hypoxia-inducible factor-1α (HIF-1α) levels, which ultimately stimulates VEGF expression [69]. More specifically, by chelating its iron ion cofactor, DFX inhibits prolyl hydroxylase, an enzyme responsible for HIF-1α degradation [70]. In animal models, serial DFX injections improved ischemic flap survival through enhanced blood perfusion and capillary formation [69,71]. Interestingly, DFX’s increase of HIF-1-α levels seems to have a direct effect on ASCs. In vitro, DFX enhanced the survival of ASCs obtained from human lipoaspirate after exposure to antimycin, an HIF-1α inhibitor [72]. Nevertheless, a 2019 study showed no positive effect of DFX on fat graft retention in a murine model [30] (Table A2). However, in this study, grafts were only soaked for five minutes into a DFX solution before transplantation, which may not be enough to exert a positive effect. Other studies addressed this problem by using serial injections into the fat grafts after transplantation. Temiz et al. obtained 24% of fat graft weight maintenance in a murine model when treated with DFX injections every three days for a month, compared to only 8% of maintenance for control grafts treated with PBS [73]. Graft quality was also improved since DFX decreased graft fibrosis and inflammation [73]. This is in accordance with other studies showing that DFX may be able to decrease tissue fibrosis in renal tissue and irradiated wounds [74,75]. DFX also proved useful when used as a preconditioning agent at recipient sites. Kim et al. injected rat scalps with DFX every two days for a total of five treatments before performing autologous fat grafting [76]. This pretreatment led to improved adipocyte viability and microvascular density as well as increased volume retention, with DFX-treated grafts maintaining 84% of their volumes compared to only 59% for untreated grafts [76]. Another study used serial DFX injections for site preconditioning on irradiated mice scalps before performing fat grafting using human lipoaspirate. DFX pretreatment not only increased volume retention (71.75% compared to 49.47% for controls), but it also improved blood perfusion, as measured using laser Doppler analysis [77]. DFX can thereby help counteract radiation-induced hypovascularity, which is particularly pertinent for women undergoing breast reconstruction after cancer treatment. Importantly, no studies have reported an increased oncological risk with DFX administration [77]. On the contrary, many studies showed an anti-tumor effect [78,79] as well as a sensitivity of many breast cancer types to iron chelation [80]. A remaining issue is that repeated injections of DFX may be complicated in a clinical setting and could cause discomfort for patients. However, transdermal drug delivery of DFX has been used in many studies and has shown success in improving skin wound healing in diabetic [81] and chronically irradiated mice [82]. Another study used DFX chitosan-hyaluronic acid microspheres to achieve a sustained release of DFX over 10 days, which led to increased angiogenesis using an in vitro model [83]. Thus, DFX seems to have good potential for clinical applications, especially considering its safe use in patients with chronic iron overload [84]. A synergistic effect by preconditioning both the fat graft and the recipient site could be possible and should also be investigated. Finally, dosage also seems to be an important aspect, since Lin et al. showed that higher concentrations of DFX had less of an impact on graft weight and volume retention [85]. Thus, dosage should also be studied and optimized.

#### 3.2.4. Basic Fibroblast Growth Factor (bFGF)

bFGF, also known as FGF-2 or FGF-β, has been proven to be a potent stimulator of angiogenesis [86,87]. It also seems that bFGF could induce adipogenesis in vivo. Indeed, Tabata et al. showed de novo adipose tissue formation when bFGF mixed with an extract of basement membrane protein (Matrigel^TM^) was injected subcutaneously into mice, while Matrigel^TM^ alone had no effect [88]. Paradoxically, bFGF has also been reported to suppress pro-adipogenic genes [89] and the adipogenic differentiation of adipose precursors [90,91]. It has thus been suggested that bFGF’s positive impact on adipogenesis is due to its effect on neovascularization rather than a direct impact on adipocytes. In fact, angiogenesis has been proven to not only promote adipose precursor migration but also their proliferation and adipogenic differentiation through the molecular environment secreted by endothelial cells [92,93,94]. Kawaguchi et al. showed that when Matrigel^TM^ was injected subcutaneously with bFGF, increased vascularization led endogenous precursor cells to migrate to the injection site within the first week after transplantation [93]. Adipose differentiation of precursor cells subsequently followed [93]. However, bFGF’s half-life remains an obstacle. Some studies still showed a positive effect of bFGF on graft retention when used in its free form, but the growth factor was mixed with insulin [28], which might explain the effect on graft maintenance (Table A2). Animal studies using fragmin/protamine microspheres [95] and gelatin microspheres [96] containing bFGF all showed increased graft retention and vascularization when compared to controls. Mixing of bFGF microspheres with fat grafts in a canine model also showed that bFGF induced the development of new adipocytes while preventing cell atrophy [96]. Following these findings, a 2015 clinical trial used a collagen sponge and bFGF-loaded PLGA microspheres in vocal cord fat grafts, showing an improvement in graft maintenance and good clinical potential of the growth factor [97].

On the other hand, Yuksel et al. showed that fibrosis was more prominent in grafts containing bFGF PLGA microspheres compared to grafts treated with other growth factors, such as insulin [29]. This result is corroborated by other studies using bFGF dextran microspheres [98,99,100]. It is, however, important to note that dextran microspheres do not degrade in vivo [100], which could lead to a foreign body reaction and thus cause more aberrant collagen formation. Regardless, bFGF seems to act as a cell activator able to increase collagen matrix production [101]. In vitro, adding bFGF to human lipoaspirate resulted in enhanced activity and hyperproliferation of fibroblasts, leading to more collagen production [101]. Thus, bFGF should be used with caution in tissue regeneration to avoid fibrous ingrowth. In fact, dose–response curves should be investigated to find optimal concentrations of bFGF, since studies showed that higher doses could lead to increased inflammation and fibrosis [88]. Still, the literature suggests that more fibrous grafts can be useful in specific cases, since increased density could be judicious in some situations, such as supraperiosteal augmentation of the facial skeleton, for example [29].

#### 3.2.5. Botulinum Toxin A (BTX)

BTX is a neurotoxin produced by the anaerobic bacterium *Clostridium botulinum*. Through its inhibition of acetylcholine release, it leads to muscle paralysis via chemodenervation of the motor end plate [102]. This paralyzing effect is particularly interesting for fat graft maintenance since muscle movement could decrease fat survival after transplantation [103]. Indeed, it has been suggested that the mechanical forces that occur with mobilization inhibit the sprouting of new blood vessels, reducing vascularization and increasing ischemia [104]. In addition, mechanical forces are also associated with the inhibition of adipogenesis [105]. Hence, Cho et al. showed that adipogenesis and vascularization were both improved with a mechanically stable environment in a murine model [106]. It was thereby hypothesized that decreasing muscle movement could improve fat graft maintenance. A clinical trial conducted by Liu et al. in 2024 showed that mixing autologous lipoaspirate with BTX before injecting it into breasts resulted in significantly higher volume retention compared to lipoaspirate alone (16.43% and 9.62% higher retention at 3 and 6 months, respectively) [107] (Table A2). Similarly, murine studies mixing BTX with autologous fat before injection resulted in a significantly higher volume and weight retention of grafts when compared to controls [108,109]. Similar results were obtained when fat pretreated with BTX was injected into rabbit ears and compared with untreated grafts [110]. However, these studies did not investigate whether the BTX-induced improvement was due to muscle paralysis or if BTX had other possible effects. Wu et al., therefore, conducted a similar experiment by performing supramuscular fat graft on mice hindlimbs, adding BTX to the mixture or not, and analyzing limb movement using a gait analysis system [111]. In addition to improved graft retention rates and vascularization, limb paralysis was obtained in the BTX group, suggesting that the toxin’s positive effect is at least partly due to its induction of temporary paralysis, which lasted up to eight weeks [111]. These results support data from a previous study, during which femoral nerve sectioning improved fat graft retention in a rat model [112]. Similarly to DFX use, site preconditioning also represents an interesting approach with BTX. Wu et al. showed that, at similar doses, injection of BTX into the recipient site a week before grafting gave similar results to mixing the toxin directly with the graft [111]. Interestingly, Shi et al. revealed that BTX not only supported and enhanced vascularization while reducing fibrosis of intramuscular and subcutaneous grafts, but it even improved the retention of intramuscular fat grafts [113]. This is particularly pertinent since adipose tissue injected into muscles has lower survival rates [103]. Thereby, BTX could open a new avenue for fat grafting, especially since some authors suggest that muscle tissue could be the best recipient site because of its high vasculature [114,115]. Globally, BTX represents an interesting option to improve graft maintenance and is easily applicable in a clinical setting since the concomitant use of fat grafts and BTX has safely been used in humans for the treatment of face aging [116] and facial corrections [117].

#### 3.2.6. Thymosin Beta 4 (TB4)

Thymosin Beta 4 is a peptide that has been shown to promote vascularization. In vivo, it can stimulate endothelial cell migration and tube formation, thus promoting neovascularization in limb ischemia [118]. TB4 might also have a direct effect on ASCs. In vitro, it successfully improved ASC proliferation and migration [119,120]. Interestingly, TB4 also upregulated angiogenic gene expression in ASCs in vivo, thus promoting their endothelial differentiation potential and leading to neovascularization in an ischemic limb model [121]. In 2020, Qu et al. studied the impact of this peptide on autologous fat graft retention in rabbits (Table A2). The grafts mixed with TB4 (0.005 mg/mL or 0.010 mg/mL) had significantly better weight retention compared to the control grafts mixed with saline [122]. The study also showed a dose-dependent response, with the 0.010 mg/mL dose giving better graft retention. Immunohistochemical staining for CD31 showed more vessels in the TB4 groups at all time points. Although this molecule has yet to be extensively studied, TB4 shows promise in promoting graft retention through the promotion of vascularization and should be investigated further.

### 3.3. Reducing Oxidative Stress

The *Graft replacement theory* suggests that graft volume maintenance depends mainly on the regeneration of fat tissue by cells both from the recipient site and the graft itself [13]. Hence, directly reducing oxidative stress in the graft could promote graft maintenance through enhanced cell survival and/or regeneration. Thus, a growing number of studies explore this therapeutic avenue using antioxidant molecules to improve graft retention (Table A3, Appendix C).

#### 3.3.1. N-Acetylcysteine (NAC)

NAC is an amino acid and antioxidant that decreases oxidative stress by reducing free radicals [123]. As stated above, following insufficient vascularization in the early stages after injection, fat grafts enter an ischemic state, with the production of reactive oxygen species (ROS) leading to oxidative damage. In contrast to VEGF or EPO acting indirectly on oxidative stress by improving vascularization, NAC could help by directly tackling ROS [123]. NAC is safely used in humans for many clinical applications, such as the prevention of chronic obstructive pulmonary disease and the treatment of acetaminophen-induced hepatotoxicity [124,125]. In vitro, Gillis et al. showed that NAC had a protective effect on ASCs undergoing oxidative stress after exposition to hydrogen peroxide [123]. NAC supplementation in culture, however, stopped ASC differentiation into mature adipocytes while increasing their proliferation rate [123]. When added to the tumescent solution used during autologous fat grafting in a rat model, NAC significantly increased fat graft retention from 17% to 46% and led to reduced fibrosis, inflammation, and a 33% increase in adipocyte density when compared to a regular tumescent solution [123] (Table A3). There was, however, no significant difference in vascularization [123]. Pietruski et al. had a similar experience in a clinical trial with 15 patients and obtained a 12.9% increase in the retention rate when using a NAC tumescent solution [126]. There was no difference in the expression of angiogenic genes, which is consistent with the results of a previous study showing that NAC has little effect on vascularization [123]. It is important to note that ROS plays an important role in adipogenesis by inducing the differentiation of adipogenic progenitors into mature adipocytes [127]. A potential explanation of NAC’s positive effect on graft retention is that it initially decreases oxidative stress, which prevents ASCs’ differentiation and increases their proliferation. When the effect of NAC attenuates, the augmentation of ROS could stimulate the differentiation of a now higher quantity of ASCs, which could improve graft maintenance [123]. Thus, NAC represents a safe and promising agent for improving fat graft retention. However, further clinical trials with larger groups of patients would be pertinent.

#### 3.3.2. Other Antioxidants of Interest

Despite being less studied, some molecules feature good potential for increasing fat graft maintenance and should be discussed (Table A3). Melatonin, a hormone synthesized in the pineal gland, serves as an antioxidant by decreasing the production of ROS [128] and neutralizing free radicals [129]. Melatonin also regulates signaling pathways involved in MSC differentiation [130]. In vitro, melatonin protected ASCs against oxidative stress and decreased apoptosis following exposition to hydrogen peroxide [131]. Pretreatment of ASCs with melatonin also significantly increased their proliferation, the expression of prosurvival signaling pathways P-Erk1/2 and P-Akt, and the levels of the antioxidative enzymes catalase and heme oxygenase (HO)-1 [132]. Melatonin also increased fat browning and macrophage activation of fat grafts in a murine model, suggesting a possible additional mechanism of action [133]. However, high doses of melatonin may be ineffective in graft pretreatment since Dang et al. showed that 20 mg/Kg of melatonin was able to improve weight retention and reduce inflammation and fibrosis, while 40 mg/Kg had no impact [133].

Similarly, vitamin E, an antioxidant vitamin, has been reported to reduce adipose tissue oxidative stress and inflammation [134]. It even decreased radiation-induced fibrosis in many studies, including breast cancer patients [135,136]. Using a murine model, mixing vitamin E with human lipoaspirate improved volume retention of grafts while also decreasing inflammatory cytokines levels [137]. It also increased VEGF levels, which supports other studies stating that vitamin E can enhance tissue vascularity [138]. Vitamin E also seems effective when administered orally, with Cinar et al. showing improved graft maintenance in rats following daily orogastric gavage of vitamin E and C [139]. In the same study, zinc, another antioxidant, had similar effects to vitamin E in graft retention [139]. Comparable effects were seen with vitamin D (calcitriol), for which the systemic and sustained administration of calcitriol improved the volume and weight retention of fat grafts in mice [140]. Moreover, vitamin D also increased mitochondrial activity in adipocytes undergoing hypoxia in vitro [140]. Interestingly, incubation of fat with calcitriol before transplantation did not have an impact on graft retention, showing once again the importance of half-life consideration in the preconditioning of lipoaspirate [140].

Another promising antioxidant is apocynin, a natural agent found in the roots of plants such as *Apocynum cannabinum* [141]. Apocynin acts by inhibiting the activity of NADPH oxidase, a major source of ROS [142], thereby decreasing oxidative stress and cell apoptosis [143]. Apocynin has been a potential agent for the treatment of various conditions associated with free radicals, such as Parkinson’s [144] and cardiovascular diseases [145]. In a murine model, daily injections of apocynin at the recipient site for 14 days after fat grafting resulted in better cell viability and increased volume retention (58.6% vs. 22.7% for controls) [146]. However, in this study, fat was transplanted en bloc instead of being passed through a cannula, like when performed in a clinical setting. Further studies with a more standard fat grafting procedure are needed.

Finally, berberine (BBR) is a known antioxidant that has been shown to reduce oxidative stress in smooth muscles and mesangial cells [147]. In an in vitro study, BBR improved ASC viability and decreased apoptosis under nutrient-deficient conditions [148]. In vitro, BBR also decreased ROS production by ASCs and regulated their autophagy and apoptosis [149]. In a murine model, the molecule significantly improved the volume and weight retention of fat grafts when they were soaked in a BBR solution and then injected with the same solution daily for seven days [149]. These antioxidant effects can thus be beneficial for fat grafting and should be further investigated.

## 4. Conclusions

In conclusion, several soluble mediators have the potential to improve the outcomes of fat grafting. While current protocols might remain novel and suboptimal, preconditioning strategies are more readily accessible than other modalities, such as those including gene transfer or the cell enrichment of fat grafts. Indeed, cell-assisted lipotransfer (CAL) techniques using ASCs or the autologous SVF fraction have been highly investigated in recent years [5] and recently reviewed [150,151]. Even though these therapies are beyond the focus of the current review, their importance in improving cellular regeneration and graft retention is now recognized by many teams [151]. ASCs are not only precursor cells able to generate mature adipocytes but are also endowed with attractive secretory properties of therapeutic molecules (such as endogenous proangiogenic factors), in addition to their ability to act as pericytes and thus regulate the capillary’s size [150]. Many studies combined SVF or ASCs with soluble factors, namely, melatonin [152], bFGF [106,153], angiogenin-1 [51], and VEGF [51,56]. These studies combined the sustained delivery of the molecules with ASCs, using various strategies such as microspheres and a fibrin matrix. The molecular and cellular treatments not only showed improved graft retention but a potential synergistic effect. Other teams used CAL to ensure the sustained delivery of molecules such as VEGF using gene therapy [31,154,155]. Indeed, VEGF-transfected MSCs have been used in many pioneering studies to increase VEGF expression, vascularization, and graft volume retention [31,154,155]. The crucial roles of various pro-angiogenic therapies applied to fat grafting were recently reviewed, also examining the survival mechanisms that are more specific to large-volume fat grafting [11,150]. However, advanced gene or cell-based therapies are more difficult to transition to the clinic according to each country’s regulatory process. These techniques show great promise in improving graft maintenance and have the advantage of not relying on effective delivery systems or carriers to ensure efficacy, as is the case with molecular preconditioning. However, SVF and ASC supplementation of fat grafts have other limitations. Cell processing requires harvesting more tissue and adds time to the surgical procedure, which is not the case with molecular preconditioning [151]. Moreover, the use of a cell-based therapy is more expensive, and the optimal cell concentration leading to superior results is still unknown. Most importantly, the oncological safety of cellular supplementation is not yet guaranteed. For example, stem cells from the SVF may promote the mobility, proliferation, and recurrence of breast cancer cells [151]. ASCs could also increase the local vascularization of tumors, thus promoting cancer growth [151]. Even though similar oncological risks could be present when using specific growth factors, many molecules described in this review are frequently used safely as drugs for other disease conditions.

New molecules or techniques have more recently been proposed to potentially improve graft retention through distinct mechanisms of action than the classical ones described. Namely, poloxamers such as P188 have been shown to protect the adipocyte membrane, thus reducing cellular damage and graft reabsorption. P188 successfully improved graft retention in murine models while reducing fibrosis and inflammation [156,157]. Another interesting approach consists of reducing the adipocyte size to improve the contact surface area and the number of adipocytes benefiting from nutrient diffusion [158]. A preclinical study proposed a new technique of *compact fat grafting,* during which the use of MLN924, an enzyme inhibitor controlling intracytoplasmic lipid accumulation, resulted in a reduced adipocyte size and increased graft retention [158]. The adipocyte size came back to normal within eight weeks, making this an interesting strategy for graft improvement without the risk and costs associated with growth factor usage [158]. Finally, molecules such as quercetin seemed to favor fat graft maintenance by promoting browning of adipose tissue [159], a mechanism also suggested for melatonin [133]. Beige or brown adipocytes usually feature a smaller volume and higher tolerance to a hypoxic microenvironment. Moreover, the browning of transplanted white fat has been considered as an adaptive response of the graft associated with better survival [150].

There is no doubt that several avenues show great potential to overcome the challenge of graft volume retention. Molecular preconditioning holds great promise, and further studies should be conducted. The key to finding a reliable and efficient molecular-based treatment lies in ways to ensure sustained delivery of the molecules. Dosage optimization and the design of studies that are more homogenous between research centers are needed to reach this goal, in addition to the development of rigorous clinical trials. Importantly, the safety of the molecules should be first confirmed, especially for growth factors that may present an oncologic risk [160]. Many mechanisms favoring graft survival are discussed in this review, but clearly, the future might lie in the investigation of combination therapies with expected synergistic effects during lipotransfer procedures.

## Data Availability

Not applicable.

## Appendix A

**Table A1:** Molecules acting through stimulation of adipogenesis.

Model	Reference	Injection Site	Graft Treatment	Graft Analysis	Findings
**PDGF**					
Human patients	Fontdevila et al. (2014) [17]	Cheeks (various planes)	A total of 2 groups of HIV patients suffering from facial lipodystrophy. Autologous lipoaspirate mixed with PRP rich in PDGF (n = 29 patients) or left untreated (n = 20 patients). Volumes were specific for each patient.	Computed tomography before grafting and 2 and 12 months after grafting. Complications after the graft were assessed (edema, ecchymosis, nodules, etc.).	PRP and PDGF did not significantly increase volume retention of fat grafts.
Rodent models	Craft et al. (2007) [22]	Scalp (subcutaneous)	A total of 3 groups (n = 8 mice/group). A total of 1 mL of human fat was left untreated or mixed with free PDGF, PDGF bound to gelatin microspheres, or blank gelatin microspheres.	Grafts were weighed 12 weeks after injection. Histological examination using Masson’s Trichrome. Immunohistochemistry using S-100 as biomarker of cell differentiation.	PDGF delivery with microspheres promoted maintenance of adipocyte architecture and weight retention compared to all groups. PDGF inhibited degene-ration into cysts and fibrous tissue.
**INSULIN**					
Human patients	Cervelli et al. (2012) [35]	Different zones of soft tissue defect depending on the patient	A total of n = 39 patients. Autologous lipoaspirate mixed with variable volumes of PRP. Volumes of lipoaspirate varied between patients. A total of 10 patients were locally injected with insulin 7 and 15 days after fat grafting.	Volume maintenance of grafts was assessed with subjective observations. Objective analysis of images. In vitro analysis on human ASCs treated with insulin, PRP, or both. Proliferation and adipogenesis were measured (bromide assay and ORO staining) in vitro.	Visually, injection of insulin increased volume maintenance when compared to grafts with PRP alone. PRP stimulated ASC proliferation in a dose-dependent manner. Insulin reduced ASC proliferation in a dose-dependent manner in vitro. When added to insulin, PRP prevented ASC proliferative arrest and improved insulin-induced lipid accumulation.
Rodent models	Olaru et al. (2020) [36]	Dorsum (subcutaneous)	A total of 4 groups (n = 6 rats/group). A total of 1 mL of fat alone or mixed with EPO, insulin, or a mix of insulin + EPO.	Grafts assessed using liquid overflow method after 2 weeks and 1 and 2 months. H&E staining, Goldner–Szekely (GS) trichrome, anti-CD68, anti-vimentin, and anti-CK labelings.	Insulin improved graft survival and quality compared to control. Combination of insulin and EPO had better outcomes on vascularization and survival than each growth factor alone. Retention rates after 2 months: ○ Fat alone: 35%. ○ EPO: 85%. ○ Insulin: 75%. ○ EPO + insulin: 95%.
	Okyay et al. (2019) [30]	Dorsum. A total of 4 injections, 1 site for each treatment (subcutaneous)	A total of 4 groups (n = 22 rats). A total of 1 mL of autologous fat injected after being incubated for 5 min in a solution of insulin, metoprolol, deferoxamine (DFX), or PBS.	Grafts were weighed 3 months post-grafting. H&E staining, anti-CD34, anti-CD31, and anti-perilipin labelings.	Regarding graft retention, insulin, metoprolol, and DFX treatments did not significantly differ from control grafts (PBS). Angiogenesis (CD31 labeling) was significantly higher in DFX and insulin groups, but there was no significant difference between insulin and DFX.
	Hong et al. (2010) [28]	Dorsum. A total of 4 injections, 1 site for each treatment (subcutaneous)	A total of 4 groups (n = 24 rabbits). A total of 2 mg of autologous fat injected after being soaked for 5 min in PBS, DMEM, DMEM + insulin, or DMEM + insulin + bFGF.	Rabbits euthanized after 1, 3, 6, and 12 months post-injection. Fat grafts were weighed. H&E staining.	Pretreatments containing insulin or bFGF resulted in significantly greater survival of grafts at 6 months and 1 year (41% survival for insulin group vs. 41.5% for bFGF group and 15% for PBS group). Insulin and insulin + bFGF reduced cyst formation and fibrosis and increased capillary density. No significant difference between insulin alone and insulin + bFGF.
	Lu et al. (2009) [31]	Dorsum. A total of 4 injections, 1 site for each treatment (subcutaneous)	A total of 4 groups (n = 18 mice). A total of 0.3 mL of human lipoaspirate was mixed with adenovirally VEGF transduced ASCs, control ASCs, insulin, or DMEM.	Volume of grafts assessed with the liquid overflow method after 6 months. H&E staining and CD31 labeling.	Insulin treatment did not substantially improve the outcome of autologous fat grafts: higher weight than control, but more fibrosis, inflammation, and necrosis.
	Ayhan et al. (2001) [32]	Dorsum. A total of 2 injections, 1 site for each treatment (supramuscular)	A total of 2 groups (n = 10/group). Group 1: fat graft treated with PBS on one side and insulin on the other. Group 2: fat graft treated with PBS on one side and metapyrolol on the other.	Volume of grafts was assessed using liquid overflow method after 9 months. H&E staining.	Significantly lower resorption in insulin group compared to PBS. Insulin-treated grafts had considerable fibrosis, inflammation, fat necrosis, and mature adipocytes. Significantly lower resorption and more surviving adipose tissue in grafts treated with metapyrolol when compared to insulin grafts or PBS grafts.
	Yuksel et al. (1999) [29]	Dorsum (under the *panniculus carnosus*)	A total of 7 groups (n = 6 mice/group). A total of 500 mg of autologous fat left untreated or mixed with PLGA containing different growth factors: insulin or IGF-1 or bFGF or insulin + IGF-1 or insulin + IGF-1 + bFGF or empty.	Grafts were weighed after 12 weeks. H&E staining.	Long-term local delivery of growth factors by PLGA microspheres improved fat graft survival when compared to controls: ○ Insulin: 79.9%. ○ IGF-1: 87.5%. ○ bFGF: 72.9%. ○ Insulin + IGF-1: 87.7%. ○ Insulin + IGF-1 + bFGF: 68.2%. ○ Blank spheres: 76.7%. ○ Fat alone: 77.3%. Absence of significant difference in graft survival between treated groups.
	Moscona et al. (1994) [33]	Cheeks (subcutaneous)	Four groups (n = 3 rats/group): A total of 3 to 4 mL of autologous fat mixed with insulin. A total of 3 to 4 mL of autologous fat for 15 min in insulin solution. A total of 3 to 4 mL of autologous fat without treatment. A total of 3 to 4 mL of autologous fat soaked 15 min in control solution (buffer solution).	Histological analysis using a semi-quantitative scale at 12 weeks.	No significant difference in histological characteristics: cell architecture, neovascularization, and inflammation. Fibrosis was more prominent in untreated fat compared to other groups.
	Nguyen et al. (1990) [34]	Ear (subcutaneous) and rectus muscle (intramuscular)	A total of 2 groups (n = 28 rabbits). A total of 3 to 4 mL of autologous fat mixed with insulin and injected into the same animal (ear and rectus muscle). The other side was used as control and injected with untreated fat.	Biopsy specimens taken at 2 weeks and after 1, 2, 4, 6, and 9 months post-injection. H&E and Wilder’s silver stainings	No histopathologic differences in the adipocytes transplanted with or without insulin in all examined grafts.
**BETA-BLOCKERS (Metoprolol/Metapyrolol)**					
Rodent models	Okyay et al. (2023) [39]	Dorsum. A total of 4 injections, 1 site for each treatment (subcutaneous)	A total of 4 groups (n = 10 rats). A total of 0.5 g of autologous fat injected after being incubated for 5 min in a solution as follows: Group 0: 0.9% sodium chloride. Group 1: 1 mg/mL of metoprolol. Group 2: 2 mg/mL of metoprolol. Group 3: 3 mg/mL of metoprolol.	H&E and Masson Trichrome stainings at 3 months post-grafting. Immunohistochemical examination by FGF-2 and perilipin labelings.	As the metoprolol dose increased, so did the quality and viability of the grafts. Groups 2 and 3 had significantly higher adipocyte viability and vascularization with less fibrosis. Group 3 was superior to all groups.
	Okyay et al. (2019) [30]	Dorsum. A total of 4 injections, 1 site for each treatment (subcutaneous)	A total of 4 groups (n = 22 rats). A total of 1 mL of autologous fat injected after being incubated for 5 min in a solution of insulin, metoprolol, deferoxamine, or PBS.	Grafts weighed after 3 months. H&E staining, anti-CD34, anti-CD31, and anti-perilipin labelings.	Insulin, metoprolol, and DFX grafts did not differ significantly from control grafts (PBS). No significant impact of metoprolol on angiogenesis.
	Ayhan et al. (2001) [32]	Dorsum. A total of 2 injections, 1 site for each treatment (supramuscular)	A total of 2 groups (n = 10 rats/group). Group 1: Fat graft treated with PBS on one side and insulin on the other. Group 2: Fat graft treated with PBS on one side and metapyrolol on the other.	Volume of grafts assessed using liquid overflow method after 9 months. H&E staining.	Significantly lower resorption in insulin group compared to PBS. Significantly lower resorption and more surviving adipose tissue in grafts treated with metapyrolol when compared to insulin or PBS grafts.
**SDF-1**					
Rodent models	Hamed et al. (2012) [43]	Scalp (subcutaneous)	A total of 3 groups (n = 10 mice/group) A total of 1 mL of autologous fat mixed with PBS, SDF-1, or monoclonal antibody against SDF-1 and injected into diabetic mice.	Grafts weighed and volumes measured using the liquid overflow method after 15 weeks. H&E staining.	Locally delivered SDF-1 increased fat graft retention (same survival as non-diabetic mice). SDF-1 increased neovascularization and reduced fat cell apoptosis.
**INDOMETHACIN**					
Rodent models	Zhan et al. (2017) [45]	Dorsum (subcutaneous)	A total of 4 groups (n = 5 mice/group): A: Autologous fat alone. B: Autologous fat + SVF (10,000 cells). C: Autologous fat + 200 mM of indomethacin. D: Autologous fat + 200 mM of indomethacin + SVF (10,000 cells).	Volume assessed using the liquid overflow method after 2, 4, and 12 weeks. H&E staining, anti-CD31, and anti-perilipin labelings.	The indomethacin and SVF groups all had superior volume retention compared to control at each time points. Retention at 12 weeks: ○ A: 14.9%. ○ B: 25.7%. ○ C: 28.8%. ○ D: 35.76%. Indomethacin groups had better cell viability, but no effect on vascularization.
**SALVIANOLIC ACID B (SAL-B)**					
Rodent models	Sun et al. (2023) [48]	Dorsum (subcutaneous)	A total of 3 groups (n = 15 mice/group): A: 0.2 mL of human lipoaspirate + injection of 0.2 mL of saline every 2 days. B: 0.2 mL of human lipoaspirate + injection of 0.2 mL of Sal-B at 10 mM/L every 2 days. C: 0.2 mL of human lipoaspirate + injection of 0.2 mL of Sal-B at 50 mM/L every 2 days.	Grafts weighed and volume assessed using the liquid overflow method after 2, 4, and 12 weeks. Microcomputed tomography (Micro-CT) at each time point. H&E staining, anti-CD11, anti-CD206, and anti-perilipin labelings.	Sal-B groups had significantly better retention compared to the control at 12 weeks. Sal-B at 50 mM/L had the best results. Sal-B groups had lower fibrosis and inflammatory cell infiltration. Adipocyte viability was increased in treatment groups.
	Sun et al. (2021) [47]	Dorsum (subcutaneous). The same injection on both sides	A total of 3 groups (n = 6 mice/group): A: 0.2 mL of human lipoaspirate + injection of 0.2 mL of saline every 2 days. B: 0.2 mL of human lipoaspirate + injection of 0.2 mL of Sal-B at 10 mM/L every 2 days. C: 0.2 mL of human lipoaspirate + injection of 0.2 mL of Sal-B at 50 mM/L every 2 days.	Grafts on the left side were harvested at 14 days. They were assessed using H&E staining and anti-perilipin labeling. Grafts on the right side were scanned by micro-CT then weighed, and their volume was assessed using the liquid overflow method. H&E staining and anti-perilipin labeling.	At 28 days, the 50 mM/L group had better adipocyte viability and less inflammatory cell infiltration. It also had significantly better retention than the control. ○ A: 14.51%. ○ B: 41.36%. ○ C: 59.36%. Sal-B group had significantly better graft retention compared to the control at 12 weeks. A total of 50 mM/L had the best results. Sal-B groups had lower fibrosis and inflammatory cell infiltration. Adipocyte viability was increased in treatment groups.

ASC: adipose-derived stem/stromal cells; Anti-CK: anti-cytokeratin; DFX: deferoxamine; DMEM: Dulbecco’s modified eagle medium; EPO: Erythropoietin; FGF/bFGF: fibroblast growth factor; H&E staining: hematoxylin and eosin staining; HIV: human immunodeficiency virus; IGF-1: insulin-like growth factor 1; Micro-CT: microcomputed tomography; ORO: Oil Red O; PBS: phosphate-buffered saline; PDGF: platelet-derived growth factor; PRP: platelet-rich plasma; Sal-B: salvianolic acid B; SDF-1: Stromal-cell derived factor 1; VEGF: vascular endothelial growth factor.

## Appendix B

**Table A2:** Molecules acting through improvement in vascularization.

Model	Reference	Injection Site	Graft Treatment	Graft Analysis	Findings
**VASCULAR ENDOTHELIAL GROWTH FACTOR (VEGF)**					
Rodent models	Zhang et al. (2021) [161]	Dorsum. A total of 4 injections, 1 site for each treatment (subcutaneous)	A total of 4 treatments (n = 12 mice/treatment). A total of 0.8 mL of human lipoaspirate mixed with different ratios of Liquid Phase Concentrated Growth Factors (LPCGFs) or different ratios of PRP.	Grafts weighed after 15, 30, 45, and 60 days. H&E staining and immunohistochemical labeling.	Addition of LPCGFs to fat grafts reduced their absorption by 5 to 15%. Significant increase in vessels, VEGF, and TGF-β when compared to PRP and control. Higher proportions of LPCGFs provided better results.
	Ding et al. (2015) [59]	Dorsum. A total of 4 injections, 1 site for each treatment (subcutaneous)	A total of 4 groups (n = 24 mice). A total of 0.2 mL of human lipoaspirate mixed with VEGF calcium alginate (CA) microspheres, empty CA microspheres, free VEGF, or DMEM.	Grafts weighed after 3, 6, and 12 weeks. Grafts from weeks 3 and 6 observed using electron microscopy. H&E staining and CD34 labeling.	CA microspheres loaded with VEGF significantly increased weight retention (29.6 mg at 12th week vs. 20.5 mg for empty microspheres and 6.5 mg for DMEM group). VEGF CA microspheres promoted vascularization. Free VEGF barely exerted its biological function when applied directly.
	Zhang et al. (2014) [57]	Dorsum. Four injections; one site for each treatment (subcutaneous)	A total of 4 groups (n = 28 mice). A total of 0.2 mL of human lipoaspirate mixed with VEGF chitosan nanospheres, empty nanospheres, free VEGF, or DMEM.	Mice euthanized at weeks 1, 3, 6, and 12. Grafts were weighed and VEGF content quantified using ELISA at week 1. H&E staining and CD34 labeling. Electron microscopy. In vitro studies were conducted to determine toxicity of nanospheres.	Cell viability significantly decreased by chitosan nanospheres at higher concentrations (100 mg/mL). VEGF chitosan nanospheres significantly promoted fat graft neovascularization and adipocyte survival. VEGF nanospheres significantly increased weight survival on the 12th week. Free VEGF had no significant impact compared to controls.
	Topcu et al. (2012) [58]	Dorsum (under the *panniculus carnosus*)	A total of 4 groups (n = 6 mice/group) A total of 0.5 g of autologous fat left untreated or mixed with VEGF-enriched calcium alginate (CA) microspheres or empty CA microspheres. Another group received VEGF microsphere injection at recipient site 21 days prior to grafting.	Fat grafts were harvested and weighed after 90 days. H&E staining.	Both VEGF microspheres treatments improved weight survival compared to controls (125.3% for preconditioning and 141.6% for concomitant use). There was no significant difference between the 2 VEGF groups. VEGF microspheres improved MVD and relative adipocyte indexes. Empty microspheres also improved graft weight survival, which may be due to higher fibrosis.
	Chung et al. (2012) [55]	Flank (subcutaneous)	A total of 3 groups (n = 6 mice/group). A total of 1 mL of human lipoaspirate untreated or mixed with VEGF-loaded PLGA microspheres or empty microspheres.	Grafts were weighed and volumes assessed using liquid overflow method after 3 and 6 weeks. H&E staining and human CD31^+^ immunolabeling.	VEGF microspheres significantly improved graft sustainment (weights and volumes). VEGF improved the number of blood vessels that were formed by human endothelial-derived cells.
	Hamed et al. (2010) [54]	Scalp (subcutaneous)	First experiment A total of 3 groups (n = 10 mice/group). A total of 1 mL of human lipoaspirate mixed with PBS, EPO, or VEGF. Grafts then received injections of the same additive every 3 days for 18 days. Second experiment A total of 2 groups (n = 10 mice/group). Untreated fat grafts received PBS or VEGF injections every 3 days for 18 days.	Grafts were weighed, and volume was assessed using liquid overflow method after 15 weeks. H&E staining, anti-CD31 and anti-CD68 labelings. Western blot analysis was used to quantify angiogenic and apoptotic factors.	Treatment with free VEGF had no effect on graft survival. VEGF did not exert an anti-apoptotic effect on the fat grafts.
	Lei et al. (2008) [162]	Dorsum (subcutaneous)	A total of 3 groups (n = 16 rats/group). After injection of 0.2 g of autologous fat, recipient sites were injected with normal saline, plasmid DNA encoding rhVEGF, or control plasmid DNA.	Grafts were harvested and weighed at 3, 7, 15, and 30 days after grafting. H&E staining and immunohistochemical labeling. VEGF expression was assessed using Western blot.	VEGF significantly improved graft retention after 7, 15, and 30 days. Plasmid encoding VEGF induced significantly higher expression of VEGF and angiogenesis in fat grafts.
**ERYTHROPOIETIN (EPO)**					
Rodent models	Olaru et al. (2020) [36]	Dorsum (subcutaneous)	A total of 4 groups (n = 6 rats/group). A total of 1 mL of fat alone or mixed with EPO, insulin or insulin + EPO.	Graft volume was assessed using liquid overflow method after 2 weeks, 1 month, and 2 months. H&E staining, GS trichrome, anti-CD68, anti-vimentin, and anti-CK labelings.	Combination of insulin and EPO had better outcomes regarding vascularization and survival than each growth factor alone. EPO had a better overall effect than insulin on vascularization and graft maintenance. Retention rates after 2 months: ○ Fat alone: 35%. ○ EPO: 85%. ○ Insulin: 75%. ○ EPO + insulin: 95%.
	Hamed et al. (2010) [54]	Scalp (subcutaneous)	First experiment A total of 3 groups (n = 10 mice/group) A total of 1 mL of human lipoaspirate mixed with PBS, low-dose EPO (1000 IU/kg), or high-dose EPO (5000 IU/kg). Grafts then received injections of the same additive every 3 days for 18 days. Second experiment A total of 2 groups (n = 10 mice/group). Untreated fat grafts receiving PBS or VEGF injections every 3 days for 18 days.	Grafts were weighed and volume assessed using liquid overflow method after 15 weeks. H&E staining, anti-CD31, and anti-CD68 labelings. Western blot analysis to quantify angiogenic and apoptotic factors.	EPO improved survival of fat graft in a dose-dependent manner (higher doses of EPO were more efficient). EPO improved angiogenesis and apoptosis rates in a dose-dependent manner.
**DEFEROXAMINE (DFX)**					
Rodent models	Lin et al. (2023) [85]	Dorsum. Four injections; one site for each treatment (subcutaneous)	A total of 4 treatments (n = 25 mice). A total of 0.3 mL of autologous fat injected after being mixed with different concentrations of DFX: A: 0 mM (mixed with PBS). B: 0.5 mM of deferoxamine. C: 1 mM of deferoxamine. D: 4 mM of deferoxamine.	Grafts were weighed after 1 and 3 months. Volume was assessed using the liquid overflow method. H&E staining, anti-CD31, and anti-perilipin labelings. Levels of *VEGF* assessed using PCR.	The DFX groups all had superior weight and volume retention compared to control. Group C had the best retention of all compared to control: ○ Volume (C vs. A): 55.75% vs. 29.78%. ○ Weight (C vs. A): 49.19% vs. 25.06%. DFX groups had higher MVD, adipocyte integrity, and VEGF expression.
	Okyay et al. (2019) [30]	Dorsum. Four injections; one site for each treatment (subcutaneous)	A total of 4 groups (n = 22 rats). A total of 0.5 g of autologous fat injected after being incubated for 5 min in a solution of insulin, metoprolol, DFX, or PBS.	Grafts were weighed after 3 months. H&E staining, anti-CD34, anti-CD31, and anti-perilipin labelings.	Regarding graft retention, insulin, metoprolol, and DFX treatments did not significantly differ from control grafts (PBS). Angiogenesis was significantly higher in deferoxamine and insulin group, with no significant difference between insulin and deferoxamine.
	Kim et al. (2019) [76]	Scalp (supramuscular)	A total of 3 groups (n = 6 rats/group). Injection site was left untreated (negative controls) or preconditioned with serial injections of DFX or saline every 2 days (5 treatments in total).	After last injection, half the rats underwent Doppler analysis to assess perfusion. The remaining rats underwent fat grafting (1 mL), and grafts were harvested and weighed after 8 weeks. Volume was assessed using liquid overflow method. H&E staining and CD31 labeling.	Rat scalps treated with DFX showed significantly increased capillary neoformation and VEGF expression but no difference in SDF-1 and HIF-1 protein expression. MVD and adipocyte viability were increased in the DFX group compared to other groups. DFX increased volume retention (84% vs. 59% for negative controls and 39.8% for saline controls). No significant difference in weights between groups.
	Flacco et al. (2018) [77]	Scalp (irradiated skin) (subcutaneous)	A total of 2 groups (n = 6 mice/group). After 6 doses of radiation to the scalp, mice either received DFX injections on the scalp every 2 days (7 doses) or PBS injections in the irradiated zone. A total of 0.2 mL of human lipoaspirate fat grafting was performed afterwards.	Laser Doppler analysis (LDA) at irradiated site to measure perfusion 24 h after each treatment and every 2 weeks after transplantation. Micro-CT imaging to assess volume of graft 4 days post-op and then at weeks 2, 4, 6, and 8. H&E staining and CD31 labeling on the 8th week.	Preconditioning of recipient site with DFX prior to fat grafting enhanced vascularization and graft survival (71.75% retention on the 8th week vs. 49.47% for the PBS group). Treatment with DFX significantly increased tissue perfusion.
	Temiz et al. (2016) [73]	Scalp (subcutaneous)	A total of 3 groups (n = 8 rats/group): A total of 0.5 g of autologous fat alone. A total of 0.5 g of autologous fat + PBS. Repeated injections of PBS every 3 days for a month. A total of 0.5 g of autologous fat + DFX. Repeated injections of DFX every 3 days for a month.	Grafts harvested and weighed after 2 months. Histological analysis via CD31 labeling and Sudan black dye. TUNEL method was used to determine apoptosis rate.	Significantly higher weights in DFX group compared to the 2 control groups. No significant difference between control groups. (24% weight retention for DFX group vs. 8% and 9% for control groups). No significant difference in apoptosis rates and vascularity between the 3 groups. Fat grafts with DFX showed significant increase in fatty tissue.
**FIBROBLAST GROWTH FACTOR (bFGF or FGF-2)**					
Human patients	Tamura et al. (2015) [97]	Vocal fold. One vocal cord for each treatment (intramuscular)	A total of 2 groups. Autologous fat alone (n = 36 patients) or mixed with a collagen sponge containing bFGF PLGA microspheres (N = 8 patients) (mean volume of injection: 0.2 mL).	Volume assessed using CT imaging (mean follow-up time of 8 months).	Decrease in injected fat volume significantly lower in cases treated with bFGF (survival rate of bFGF fat was 32.6% vs. 19.8% for untreated fat).
Canine model	Tamura et al. (2007) [96]	Vocal fold. One vocal cord for each treatment (intramuscular)	A total of 2 groups (n = 12 dogs). A total of 0.5 mL of autologous fat alone or mixed with bFGF gelatin microspheres.	Volume visually assessed using direct laryngoscopy at 8, 12, and 24 weeks (no quantitative measurement). Grafts harvested at weeks 8, 12, and 24. H&E staining. At week 8, observation using electron microscopy.	Visually, fat grafts alone showed a significant decrease in volume over time, but volume of bFGF fat grafts was maintained almost completely. At week 12, diameter of adipocytes was 50% smaller in control grafts compared to bFGF. bFGF elicited growth of new adipocytes, and grafts remained viable without atrophy or fibrosis.
Rodent models	Hong et al. (2010) [28]	Dorsum. Four injections; one site for each treatment (subcutaneous)	A total of 4 groups (n = 24 rabbits). A total of 2 mg of autologous fat injected after being soaked for 5 min in PBS, DMEM, DMEM + insulin, or DMEM + insulin + bFGF.	Fat grafts were weighed at 1, 3, 6, and 12 months. H&E staining.	Pretreatments containing insulin or bFGF resulted in significantly greater survival of grafts at 6 months and 1 year (41% survival for insulin group vs. 41.5% for bFGF group and 15% for PBS group). No significant difference between insulin alone and insulin + bFGF. Insulin and insulin + bFGF reduced cyst formation and fibrosis and increased capillary density.
	Nakamura et al. (2010) [95]	Dorsum. Two injections; one site for each treatment (subcutaneous)	A total of 2 groups (n = 48 rats). A total of 0.8 g of autologous fat mixed with empty fragmin/protamin (FP) microspheres or bFGF-loaded FP microspheres.	Rats were euthanized after 10, 20, 30, and 120 days. Qualitative assessment (no quantitative weight or volume measurement). H&E and Sudan III stainings.	Grafts with empty microspheres were significantly resorbed at 30 and 120 days, while bFGF grafts seemed minimally resorbed. Fat graft combined with FGF-2 microparticles significantly stimulated revascularization.
	Yuksel et al. (1999) [29]	Dorsum (under the *panniculus carnosus*)	A total of 7 groups (n = 6 mice/group). A total of 0.5 g of autologous fat left untreated or mixed with PLGA containing different growth factors: insulin or IGF-1 or bFGF or insulin + IGF-1 or insulin + IGF-1 + bFGF or empty.	Grafts were weighed after 12 weeks. H&E staining.	Long-term local delivery of growth factors by PLGA microspheres improved survival when compared to controls (96% for bFGF vs. 46.9% for fat alone). Absence of significant graft survival between groups. bFGF increased the amount of fibrous tissue and other non-adipocyte components compared to insulin and IGF-1.
	Eppley et al. (1992) [98]	Dorsum Four injections; one site for each treatment (subcutaneous)	A total of 4 groups (n = 40 rats). A total of 500 mg of autologous fat left untreated or mixed with blank dextran microspheres, dextran microspheres soaked in cytochrome C (control solution), or dextran microspheres soaked in bFGF solution for 1 min.	Grafts were weighed after 1 and 12 months. Histological analysis using trichrome staining.	bFGF improved weight maintenance (468.8 mg after 12 months vs. 243.8 mg for untreated grafts). bFGF stimulated the formation of fibrous ingrowth. Histologically, bFGF had no direct effect on adipocyte survival or preadipocyte differentiation. Dextran microspheres were not absorbed by the body and caused more fibrous ingrowth.
	Eppley et al. (1992) [100]	Cheeks. Two injections; one side for each treatment (subcutaneous)	A total of 2 groups (n = 20 rats). A total of 250 mg of autologous fat mixed with bFGF solution or dextran microspheres soaked in bFGF solution for 15 min.	Grafts were weighed after 1 and 6 months. Histological analysis using trichrome staining.	After 1 month, there was no significant difference in graft retention. After 6 months, asymmetrical face with significantly increased weight in microbeads group (50.8% weight loss for free bFGF grafts vs. 4.4% for microspheres side). Microspheres group had more extensive fibrous ingrowth, which appeared denser around beads. No degradation of the microbeads.
	Eppley et al. (1991) [99]	Face. Two injections; one side for each treatment (subcutaneous)	A total of 2 groups (n = 15 rats). An average of 0.3 g of autologous fat alone or mixed with dextran microspheres soaked in bFGF solution for 1 min.	Grafts were weighed after 90 days. Histological analysis using trichrome staining.	bFGF microspheres improved graft maintenance. Fat grafts alone had a 19.2% decrease in weight, while bFGF grafts had a 42.3% increase in weight. Dextran microspheres were not absorbed by the body.
**BOTULINUM TOXIN A (BTX)**					
Human patients	Liu et al. (2024) [107]	Breasts	A total of 2 groups (n = 16 women patients). One breast received autologous lipoaspirate alone, and the other breast received autologous lipoaspirate mixed with 100 U of BTX.	At 3 and 6 months postoperatively, breasts were assessed using 3D photography by a blinded plastic surgeon and using a patient satisfaction survey.	Breasts with BTX had a statistically significant superior volume retention (16.43% at 3 months and 9.62% at 6 months). Satisfaction scores by patients and plastic surgeons were significantly better for the BTX side.
Rodent models	Yoon et al. (2021) [110]	Center of the ear	A total of 4 groups (n = 10 rabbits/group). A total of 1.5 mL of human lipoaspirate mixed with saline, BTX, prostaglandin E2, or polydeoxyribonucleotides (PDRNs).	Grafts were weighed, and volume was assessed using liquid overflow method at 7, 14, 28, 42, 56, and 70 days. H&E staining and CD31 labeling.	Treatment of fat with BTX, E2, and PDRNs improved weights and volumes of grafts compared to control. Treatment of fat with BTX, E2, and PDRNs improved angiogenesis.
	Wu et al. (2020) [111]	Quadriceps (supramuscular)	A total of 0.3 mL of human lipoaspirate injected onto the surface of the right quadriceps. A total of 3 groups (n = 24 mice/group): Pre-BTX: injection of BTX on the right *quadriceps femoris* 1 week before grafting. BTX: saline injection, and a week later, injection of fat mixed BTX Control: No BTX.	Grafts were harvested and volumes assessed using liquid overflow method at 1, 4, 8, and 12 weeks. H&E staining, anti-CD31, and anti-perilipin labelings. Limb movement analysis was performed (using CatWalk XT).	Pre-BTX and BTX groups had significantly higher volume retention rates. No significant difference between BTX and pre-BTX volumes at any time point. Pre-BTX and BTX groups exhibited better angiogenesis and adipocyte survival than the control group.
	Shi et al. (2019) [113]	Limbs: *quadriceps femoris* and *gastrocnemius* (subcutaneous and intramuscular)	A total of 2 treatments (n = 12 rats). Rats received 0.2 mL of fat mixed with BTX in one limb and fat mixed with PBS on the other one. For each side, intramuscular and subcutaneous injections were performed.	Electroneuromyography was performed on limbs 1 week after surgery. Grafts were weighed and volume assessed using the liquid overflow method after 12 weeks. H&E staining and whole-mount immunofluorescence labeling for measurement of MVD and adipocyte viability.	Amplitudes of electroneuromyography were smaller for the BTX-A sides. Intramuscular BTX grafts had significantly better retention rate than controls (weights and volumes). Subcutaneous BTX side did not show a significant improvement in graft retention. Significantly higher MVD and number of mature adipocytes in BTX grafts.
	Tang et al. (2017) [108]	Dorsum. Two injections; one site for each treatment (supramuscular)	A total of 2 groups (n = 6 rats). Autologous fat (mean: 1 mL) alone or mixed with BTX. In vitro, ASCs were isolated from fat of other rats and incubated with various BTX concentrations for a day.	Grafts weighed and volume assessed with liquid overflow method after 5 weeks. H&E, Oil Red O (ORO) staining, anti-CD34, anti-VEGF, and TUNEL labelings. In vitro, adipogenic differentiation and proliferation were assessed.	Mean volume and weight of grafts were significantly higher in BTX group compared to control (1.2 mL vs. 0.9 mL and 1.2 g vs. 0.87 g). TUNEL labeling revealed less apoptosis in the BTX group. Better adipogenic differentiation, adipogenesis, and VEGF expression on BTX sides. In vitro, there was a dose-dependent increase in ASC proliferation with BTX treatment.
	Baek et al. (2012) [109]	Dorsum Two injections; one site for each treatment (supramuscular)	A total of 2 groups (n = 8 mice). A total of 0.5 mL of fat from rats mixed with BTX or PBS before injection onto mice backs.	Grafts were weighed, and volume was assessed with liquid overflow method 9 weeks after injection. H&E staining.	BTX sides had significantly higher volumes (0.37 mL vs. 0.22 mL for control) and weights compared to controls (0.35 g vs. 0.18 g). Cellular integrity was higher in BTX group.
**THYMOSIN BETA 4 (TB4)**					
Rodent models	Qu et al. (2020) [122]	Ears (subcutaneous)	A total of 3 groups (n = 6 rabbits/group). A: 1.5 mL of autologous fat + 0.005 mg/mL of TB4. B: 1.5 mL of autologous fat + 0.010 mg/mL of TB4. C: 1.5 mL of autologous fat + saline.	Grafts weighed after 2, 4, and 12 weeks. H&E staining and anti-CD31 labeling.	TB4 groups had significantly better graft retention at all time points, with 0.010 mg/mL giving the best results. TB4 groups had less inflammation and fibrosis compared to controls. TB4 groups, especially group B, had significantly better vascularization compared to controls.

BTX: Botulinum Toxin A; CA: calcium alginate; DNA: Deoxyribonucleic Acid; ELISA: Enzyme-Linked Immunosorbent Assay; HIF-1: Hypoxia-Inducible Factor-1; LDA: laser Doppler analysis; LPCGFs: Liquid Phase Concentrated Growth Factors; Micro-CT: microcomputed tomography; MVD: microvascular density; PDRNs: polydeoxyribonucleotides; PLGA: polylactic-co-glycolic acid; rhVEGF: recombinant human Vascular Endothelial Growth Factor; SVF: Stromal Vascular Fraction; TB4: Thymosin Beta 4; TUNEL: Terminal Deoxynucleotidyl Transferase.

## Appendix C

**Table A3:** Molecules decreasing oxidative stress.

Model	Reference	Injection Site	Graft Treatment	Graft Analysis	Findings
**N-ACETYLCYSTEIN (NAC)**					
Human patients	Pietruski et al. (2021) [126]	Breasts (each breast receiving one of the two treatments)	A total of 2 groups (n = 15 women). A total of 145 mL of autologous lipoaspirate harvested using normal tumescent solution in one thigh and NAC-enriched tumescent solution on the other.	MRI before and 6 months after the procedure to assess volume. Shortly after transplantation, adipose tissue samples from each graft were subjected to biochemical analysis, PCR, and flow cytometry (cell viability).	NAC grafts had 12.9% lower resorption rate than control group, but not statistically significant. Concentration and activity of superoxide dismutase (antioxidant) were significantly higher in NAC-treated grafts.
Rodent model	Gillis et al. (2015) [123]	Scalp (subcutaneous)	A total of 2 groups (n = 15 mice/group). A total of 0.2 mL of autologous fat harvested using tumescent solution with or without NAC.	Graft volume analysis via micro-CT at baseline and after 4 days and 1 and 3 months. H&E staining and CD31 labeling.	NAC treatment resulted in significantly improved fat graft retention compared to control grafts (46% vs. 17%). NAC grafts showed less fibrosis and inflammation and a 33% increase in adipocyte density compared with controls. No significant difference in vascularization.
**MELATONIN**					
Rodent model	Cinar et al. (2023) [139]	Dorsum (subcutaneous)	A total of 4 groups (n = 8 rats/group) After transplantation of autologous fat (volume varying depending on the amount of fat collected), rats underwent a specific diet every day for 3 months: (1) A total of 10 mg/Kg of melatonin via orogastric gavage. (2) A total of 2 mg/Kg of elemental zinc via orogastric gavage. (3) A total of 100 mg/Kg of vitamin C and 100 mg/Kg of vitamin E via orogastric gavage. (4) Regular diet.	Grafts weighed and volumes assessed using the liquid overflow method after 3 months. H&E staining and anti-perilipin labeling.	Adipocyte integrity, volume, and weight maintenance were significantly higher in the antioxidant groups compared to control. No significant difference between the different antioxidant treatments.
	Dang et al. (2023) [133]	Scalp (subcutaneous)	A total of 4 groups (n = 18 mice/group) After transplantation of 0.3 g of autologous fat, mice were treated every day for 2 weeks with different doses of oral melatonin. (1) High dose: 40 mg/Kg/d. (2) Medium dose: 20 mg/Kg/d. (3) Low dose: 10 mg/Kg/d. (4) Control: no melatonin.	Grafts were weighed after 2, 4, and 12 weeks. H&E staining. Immunohistochemical labeling for anti-CD31, anti-CD106 (macrophage marker), and UCP-1 cells (fat browning indicator).	After 12 weeks, graft retention was higher in the medium-dose group (41.7%), while there was no significant difference between the high-dose and control groups. Less inflammation, fibrosis, and cysts in the medium-dose group, but no significant difference between the control and high-dose groups. Higher UCP-1 and CD106 expression in medium-dose group, suggesting higher fat browning possibly mediated by macrophage activation.
**VITAMIN E**					
Rodent model	Cinar et al. (2023) [139]	Dorsum (subcutaneous)	A total of 4 groups (n = 8 rats/group). After transplantation of fat, rats underwent a specific diet every day for 3 months: (1) A total of 10 mg/Kg of melatonin via orogastric gavage. (2) A total of 2 mg/Kg of elemental zinc via orogastric gavage. (3) A total of 100 mg/Kg of vitamin C and 100 mg/Kg of vitamin E via orogastric gavage. (4) Regular diet.	Grafts were weighed and volumes assessed using the liquid overflow method. H&E staining and anti-perilipin labeling.	Adipocyte integrity, volume, and weight maintenance were significantly higher in the antioxidant groups compared to control. No significant difference between the different antioxidant treatments.
	Abbas et al. (2022) [137]	Scalp (subcutaneous)	A total of 4 groups (n = 10 mice/group): mice underwent scalp irradiation and recovered for 4 weeks. No fat grafting. Fat grafting (0.2 mL of human fat) without treatment. Fat grafting (0.2 mL of human fat) treated with vitamin E. Fat grafting (0.2 mL of human fat) treated with pentoxifylline (PTX).	Volume retention was monitored in vivo with micro-CT at weeks 0, 1, 2, 4, 6, and 8 post-grafting. After 8 weeks, Masson’s Trichrome staining, anti-CD31, and anti-isoprostane labelings. Secretome was also assessed.	Vitamin E improved graft volume retention. Vitamin E decreased inflammation and oxidative stress while increasing VEGF protein levels. Vitamin E outperformed other treatments in vascularization improvement.
**VITAMIN D (CALCITRIOL)**					
Rodent model	Loder et al. (2023) [140]	Dorsum (subcutaneous)	A total of 0.3 mL of human lipoaspirate with one of four different treatments: (A) Fat incubated in a control solution for 60 min before transplantation. (B) Fat incubated in calcitriol solution for 60 min before transplantation. (C) Systemic injection of PBS (300 μL intraperitoneally) three times weekly after transplantation. (D) Systemic injection of calcitriol (300 μL intraperitoneally) three times weekly after transplantation.	Athymic mice. Grafts were weighed after 1, 4, and 12 weeks. H&E staining, anti-perilipin, and anti-CD31 labelings. Ex vivo experiments conducted to assess calcitriol’s effect on adipocyte.	In vitro, calcitriol improved adipocyte viability and mitochondrial reserve capacity. It also improved mobilization of endothelial cells. In vivo, grafts incubated with calcitriol had less fibrosis and greater adipocyte viability, but there was no difference in weight and volume retention compared to controls at 12 weeks. In vivo, systemic and sustained delivery of calcitriol improved weight and volume retention, as well as MVD.
**APOCYNIN**					
Rodent model	Keskin et al. (2021) [146]	Scalp (subcutaneous)	A total of 3 groups (n = 7 rats/group): a total of 0.4 to 0.8 g of autologous fat. After grafting, there were 3 groups of treatment. Intraperitoneal saline injection daily for 14 days. Intraperitoneal DMSO (concentration of 20%) injection daily for 14 days. Intraperitoneal DMSO + apocynin injection daily for 14 days.	Grafts were weighed and volumes were assessed with liquid overflow method after 90 days. H&E staining.	Apocynin group had significantly higher weight and volume maintenance compared to DMSO and saline groups. Volume: ○ Apocynin: 58.60%. ○ DMSO: 27.82%. ○ Saline: 20%. Weight: ○ Apocynin: 59.08%. ○ DMSO: 31.54%. ○ Saline: 22.70%. No significant difference between groups in histological analysis for fibrosis, cyst formation, vascularization and inflammation.
**BERBERINE (BBR)**					
Rodent model	Pang et al. (2023) [149]	Dorsum and neck (subcutaneous)	A total of 2 groups (n = 10 mice/group): 0.2 mL of human lipoaspirate. Treated grafts were soaked in BBR for 10 min. After transplantation, grafts had daily injections of saline or BBR (4 mM).	Grafts were weighed and volumes assessed with liquid overflow method at 14 and 28 days. H&E staining, anti-CD31, and anti-perilipin labelings.	BBR grafts had significantly better volume and weight retention at both time points compared to saline control. BBR grafts had less fibrosis, less inflammatory cells infiltration, and more vascularization.

BBR: berberine; DMSO: dimethyl sulfoxide; MRI: Magnetic Resonance Imaging; MVD: microvascular density; NAC: N-acetylcysteine; PCR: polymerase chain reaction; PTX: pentoxifylline; UCP-1: uncoupling protein 1.
